# Cytokine Profiling in Human iPSC-Derived Dopaminergic Neuronal and Microglial Cultures

**DOI:** 10.3390/cells12212535

**Published:** 2023-10-27

**Authors:** Evelyn Knappe, Franziska Rudolph, Christine Klein, Philip Seibler

**Affiliations:** Institute of Neurogenetics, University of Lübeck, Ratzeburger Allee 160, 23562 Lübeck, Germany; evelyn.knappe@student.uni-luebeck.de (E.K.); franziska.rudolph@uni-luebeck.de (F.R.); christine.klein@uni-luebeck.de (C.K.)

**Keywords:** iPSC, dopaminergic neurons, microglia, co-culture, cytokine profiling, Parkinson’s disease

## Abstract

Aside from the degeneration of dopaminergic neurons, inflammation is a key component in the movement disorder Parkinson’s disease (PD). Microglia activation as well as elevated cytokine levels were observed in the brains of PD patients, but the specific role of microglia in the disease process is unknown. Here, we generate human cellular models by differentiating iPSCs into dopaminergic neurons and microglia. We combine these cells in co-culture to perform cytokine profiling, representing the final functional outcome of various signaling pathways. For this, we used unstimulated conditions and treatment with inflammatory stressors. Importantly, only co-cultures but not the monocultures responded to IL-1β treatment suggesting co-culture-related crosstalk. Moreover, we identified the main types of released cytokines and chemokines in this model system and found a preference for the activation of the chemotaxis pathway in response to all treatments, which informs future studies on the cell-type-specific reaction to inflammatory stimulation. Finally, we detected protein level changes in PD risk factor GPNMB upon stress in microglia, further confirming the link between PD-associated genes and inflammation in human-derived cellular models.

## 1. Introduction

Loss of dopaminergic neurons in the substantia nigra is a pathological hallmark of the movement disorder Parkinson’s disease (PD). Therefore, most PD research, thus far, focuses on the pathological mechanisms in neurons. Importantly, aside from neurodegeneration, inflammation is a key component in PD. Elevated levels of tumor necrosis factor (TNF), interleukin-1β (IL-1β), transforming growth factor beta (TGF-β), IL-6, reactive oxygen species, nitric oxide species, and pro-apoptotic proteins have been found in the substantia nigra, striatum, and cerebrospinal fluid (CSF) of patients with PD [1,2,3]. Notably, the concentration of microglia is particularly high in the substantia nigra and the inflammatory response in PD is most likely mainly conferred by microglial cells [4], which makes them an important cell type to investigate. In contrast to neurons and other brain cells, microglia derive from a myeloid progenitor pool in the yolk sac and migrate into the CNS before the blood-brain barrier is shaped. They protect the brain from injury and disease by providing support to neurons via secretion of factors, clearing of dead cells or misfolded proteins through phagocytic engulfment, and defending from infectious insults [5]. 

Two prototypic pro-inflammatory cytokines, IL-1β and TNF-α, have been revealed as important mediators of the functional effects of neuroinflammation on neurodegeneration in PD models [6]. IL-1β is considered a master regulator of neuroinflammation because it leads to the expression of adhesion molecules, immune cell infiltration, and the production of many mediators of inflammation in the CNS [7]. TNF-α has many functions, including apoptosis, cell differentiation, proliferation, survival, and inflammation. Under pathological conditions, increased levels of this cytokine overactivate microglia, which then causes neuronal damage [8]. 

Interestingly, polymorphisms in the *IL1B* and *TNF* genes have been identified as risk factors of PD and may affect the onset of disease [9]. Moreover, there are several genes linked to autosomal dominant and autosomal recessive monogenic forms of PD that indicate a role of the immune system [9]. For example, mutations in *Leucine rich repeat kinase 2* (*LRRK2*) cause the most frequent dominantly inherited form of PD. *LRRK2* encodes a kinase, which was not only shown to be important for neuronal function, but also for peripheral and central immune responses [10]. Notably, *LRRK2* expression is tightly regulated in peripheral immune cells and increases in response to microbial pathogens [9]. The upregulation of *LRRK2* in PD patient compared to control microglia [11] suggests a critical role for *LRRK2* in the functioning of the immune cells of the brain. However, the specific role of microglia and their release of cytokines in PD pathophysiology is still unresolved, and due to distinct species-specific differences of microglial properties between rodents and humans [12], there is a need for studies in human-derived cell models. Microglia can play a protective role in dopaminergic neuron survival [13] but have also been shown to exacerbate a damaging effect [14] in animal studies. The usage of induced pluripotent stem cells (iPSCs) overcomes the inaccessibility of primary cells from patients and enables the investigation of human microglia–dopaminergic neuron crosstalk.

Here, we differentiated human iPSCs into dopaminergic neurons and microglia and combined previously published differentiation protocols in a co-culture to perform cytokine profiling. The specific set of produced and released cytokines reveals the final functional outcome of various signaling pathways, and we assessed such signatures under unstimulated conditions and upon treatment with lipopolysaccharide (LPS)/interferon-γ (IFN-γ), IL-1β, or TNF-α. Moreover, we analyzed protein levels encoded by genes that have been linked to PD in response to inflammatory insult. Our functional characterization of these cultures informs studies for PD research on the culture-specific responsiveness of inflammatory stimulation and identifies the main types of released cytokines and chemokines in this in vitro model system. 

## 2. Materials and Methods

### 2.1. iPSC Lines

All iPSC lines were derived from healthy individuals. A detailed list of the iPSC lines can be found in Table S1. The iPSCs were cultured in mTeSR1 medium (STEMCELL Technologies, Vancouver, BC, Canada) on Matrigel (Corning Inc., Glendale, CA, USA)-coated plates. Cells were passaged using 0.5 mM EDTA (Sigma Aldrich, St. Louis, MO, USA) in PBS (Thermo Fisher Scientific, Waltham, MA, USA) every 4–5 days.

### 2.2. Differentiation into Dopaminergic Neurons

Dopaminergic neuronal differentiation was performed according to a previously published protocol [15] with minor modifications [16]. In brief, 1.4 × 10^6^ iPSCs were seeded onto Matrigel-coated plates in mTeSR1 medium supplemented with 10 μM Rock inhibitor Y-26732 (STEMCELL Technologies). Differentiation was initiated by adding KSR medium (Knockout DMEM (Thermo Fisher Scientific, Waltham, MA, USA), Knockout serum replacement (Thermo Fisher Scientific), 2 mM L-glutamine (Thermo Fisher Scientific), nonessential amino acids (Thermo Fisher Scientific), Penicillin/Streptavidin (PAN biotec, Aidenbach, Germany), 100 μM 2-mercaptoethanol (Thermo Fisher Scientific) with 10 mM SB431542 (Tocris, Bristol, UK) and 100 nM LDN193189 (Stemgent, Cambridge, MA, USA)). After 12 days of differentiation, cells were transferred in clusters to poly-L-ornithine (Sigma Aldrich)/laminin (Roche, Basel, Switzerland)-coated dishes. Neuronal differentiation medium (Neurobasal medium (Thermo Fisher Scientific), NeuroCult SM1 (STEMCELL Technologies), 2 mM L-glutamine, Penicillin/Streptavidin) was supplemented with 20 ng/mL Brain-derived neurotrophic factor (STEMCELL Technologies), 0.2 mM ascorbic acid (Sigma Aldrich, St. Louis, MO, USA), 20 ng/mL Glial cell line-derived neurotrophic factor (STEMCELL Technologies), 1 ng/mL TGF-β3 (Peprotech, Cranbury, NJ, USA), and 0.5 mM cAMP (Enzo Lifesciences, New York, NY, USA). The medium was changed every 2–3 days. At day 21, cells were singled by using Accutase (Thermo Fisher Scientific) and plated in drops of 50 μL with a density of 3.5 × 10^5^ cells onto dishes coated with poly-D-lysine (Sigma Aldrich)/laminin. The medium was replaced every 2–3 days until day 42 of neuronal differentiation. At this point, the differentiation factors were withdrawn from the medium.

### 2.3. Differentiation into Microglia 

Differentiation of iPSCs into microglia was performed by using previously published protocols [17,18]. iPSCs were singled with Accutase and seeded with a density of 4 × 10^6^ cells/well into a 24-well Aggrewell 800 plate (STEMCELL Technologies) [18]. The culture medium composed of mTesR1 medium was supplemented with 10 μM Rock inhibitor Y-27623 (STEMCELL Technologies), 50 ng/mL BMP4 (Peprotech), 20 ng/mL SCF (Peprotech), and 50 ng/mL VEGF (Peprotech). The embryoid bodies (EBs), formed in the Aggrewells, were collected on day 4 of differentiation using a 37 μM cell strainer and were transferred into T75 cell culture flasks. The differentiation medium composed of x-Vivo 15 medium (Lonza, Basel, Switzerland) was supplemented with 2 mM glutamine (Thermo Fisher Scientific), 50 μM 2-mercaptoethanol (Thermo Fisher Scientific), 50 U/mL Penicillin/Streptomycin, 25 ng/mL IL-3 (STEMCELL Technologies), and 100 ng/mL M-CSF (STEMCELL Technologies). The medium was changed once a week. At day 30 of differentiation, myeloid progenitor cells in suspension were collected from the culture and used for further analysis or differentiation into mature microglia in co-culture with neurons. For this purpose, progenitor cells were plated onto dopaminergic neurons (day 50 of differentiation) with a density of 4 × 10^5^ cells/24 well. Maturation medium was composed of Advanced DMEM/F12 (Thermo Fisher Scientific), N2 Supplement, 2 mM glutamine, 50 μM 2-mercaptoethanol, 50 U/mL Penicillin/Streptomycin, 100 ng/mL IL-34. Half medium changes were performed every 48 h. After 14 days, the mature co-cultures were used for further analysis.

The direct differentiation of microglia was performed according to a previously published protocol [19]. Microglial progenitor cells were seeded with a density of 1 × 10^5^ cells/cm^2^. Differentiation medium composed of Advanced DMEM/F12 medium (Thermo Fisher Scientific) was supplemented with 2 mM glutamine (Thermo Fisher Scientific), 100 ng/mL IL-34 (Peprotech), 50 ng/mL TGF-β1 (STEMCELL Technologies), and 25 ng/mL M-CSF (STEMCELL Technologies) and 10 ng/mL GM-CSF (STEMCELL Technologies). The medium was half-changed twice a week for 14 days.

### 2.4. Cytokine Release Measurements

Co-cultures, microglia, and neurons were treated with stressors to induce cytokine release into the cell culture medium: 10 ng/mL IL-1β (Peprotech), 50 ng/mL TNF-α (Peprotech), or 100 ng/mL of IFN-γ (Peprotech), and LPS (Cat. No. L8274, Sigma-Aldrich, St. Louis, MO, USA) were added to the cultures (24 h at 37 °C). Untreated samples were used to determine basal levels of cytokines. The media samples were collected, and the cells pelleted and stored at −80 °C until further analysis.

The Legendplex Human Inflammation Panel 1 assay (Biolegend, San Diego, CA, USA) was used to assess the concentration of 13 pro- and anti-inflammatory cytokines simultaneously in the cell culture media according to the manufacturer’s instructions [20]. In brief, cell culture medium, assay buffer, and mixed bead solution were added in equal amounts (25 µL each) into a 96-well of a V-bottom plate. The solution was incubated on a shaker for 2 h at room temperature (RT). After centrifugation for 5 min at 250× *g*, the supernatant was removed, and a washing step with 200 µL wash buffer was performed. Afterwards, 25 µL detection antibodies were added and incubated on a shaker for 1 h at RT. Subsequently, 25 µL SA-PE solution was added directly and incubated for 30 min while shaking at RT. The centrifugation and washing steps were repeated as described above. The pelleted beads were resuspended in 200 µL wash buffer. All measurements were performed in duplicates on the spectral flow cytometer Cytek Aurora (Cytek Biosciences, Fremont, CA, USA). IL-33 was not detected with this assay and was excluded from the analysis. The analysis was performed by using the online software developed by the manufacturer (https://legendplex.qognit.com (accessed on 24 April 2023)). After a gating process, the data of the individual samples were matched with the standard curves of the respective cytokines, enabling a direct quantitation of the cytokine content in each sample. Subsequently, the cytokine concentrations were normalized to the respective protein concentration of each sample.

### 2.5. qRT-PCR

An RNeasy Mini Kit (QIAGEN, Hilden, Germany) was used to extract RNA according to the manufacturer’s instructions. Reverse transcription of total RNA into cDNA was performed using the Maxima First Strand cDNA Synthesis Kit (Thermo Fisher Scientific) with dsDNase digest according to the manufacturer’s instructions. A total of 500 ng of total RNA was used in each reaction. Quantitative determination of gene expression levels was performed on the Lightcycler96 (Roche, Basel, Switzerland) using Maxima SYBR Green/Fluorescein qPCR Master Mix (Thermo Fisher Scientific) [21]. The primers are listed in Table S2.

### 2.6. Western Blot

Protein extraction was performed using RIPA lysis buffer (25 mM TRIS-HCl pH 7.6, 150 mM NaCl, 1% NP-40, 0.1% SDS) [22]. After 30 min incubation on ice, lysates were centrifuged at 13,000× *g* for 20 min at 4 °C, and the supernatant was transferred to a new reaction tube. The DC Protein Assay (Biorad, Hercules, CA, USA) [23] was used to measure protein concentration according to the manufacturer’s instructions. For Western blot analysis, 10 µg of protein was loaded on NuPAGE™ 4–12% Bis-Tris gels (Thermo Fisher Scientific) and transferred onto a nitrocellulose membrane. After blocking for 1 h at RT with 1% milk powder (Carl Roth, Karlsruhe, Germany) in TBST (TBS, 1% Tween-20 (Sigma Aldrich)), the membrane was incubated over night with primary antibodies diluted in 1% milk powder in TBST at 4 °C. Subsequently, the membrane was washed with TBST at RT three times for 5 min each, and the second antibody, also diluted in 1% milk powder, was added at RT at a 1:8000 dilution for 1 h [24]. All antibodies are listed in Table S3. Proteins were illustrated via Supersignal West Pico Luminescent Substrate staining solution (Thermo Fisher Scientific) according to the manufacturer’s instructions.

### 2.7. Immunofluorescence Staining

Cells were fixed with 4% paraformaldehyde (Sigma Aldrich) for 10 min at RT [22]. This was followed by three washing steps with PBS for 5 min each. Fixed cells were permeabilized and blocked with PBS containing 4% normal donkey (Sigma-Aldrich) or goat serum (Thermo Fisher Scientific), 0.1% bovine serum albumin (BSA) (Sigma-Aldrich), 0.1% Triton X-100 (AppliChem, Darmstadt, Germany), and 0.05% NaN3 (Sigma-Aldrich) for 1 h at RT. Primary antibodies (Table S3) were incubated over night at 4 °C. After three washing steps with PBS for 5 min each, secondary antibodies were incubated in PBS with 3% BSA and 0.05% NaN3 for 1 h at RT, followed by three washing steps with PBS. Finally, the samples were mounted with DAPI Fluoromount-G (Southern Biotech, Birmingham, AL, USA) on glass slides [25]. Imaging was performed using confocal laser scanning microscopes: LSM 710 (Zeiss, Jena, Germany) and Stellaris 5 (Leica, Wetzlar, Germany).

### 2.8. Flow Cytometry

To characterize microglial progenitor cells, fluorescence-activated cell sorting (FACS) was used. A total of 2 × 10^5^ cells of each line were collected from the suspension cultures and washed with PBS. Staining was performed for 45 min on ice in PBS supplemented with 2 mM EDTA and 2% Knockout serum and antibodies against the monocyte markers CD14, CD45, and CD88 (Table S3). iPSCs served as a negative control. After two washing steps with PBS, the cells were resuspended in PBS with 2 mM EDTA. The measurement was performed on a BD LSR II instrument (BD Biosciences, Macquarie Park, Australia).

### 2.9. Statistics

GraphPad Prism (Version 8, GraphPad Software, La Jolla, CA, USA) was used for statistical analysis. A one-way ANOVA or two-way ANOVA followed by multiple comparison tests were used as indicated. The *p* values are indicated in figures as * *p* < 0.05, ** *p* < 0.01, *** *p* < 0.001, **** *p* < 0.0001.

## 3. Results

### 3.1. Generation of iPSC-Derived Cultures: Dopaminergic Neurons, Microglia, and Neuron-Microglia Co-Culture

**Figure 1:**
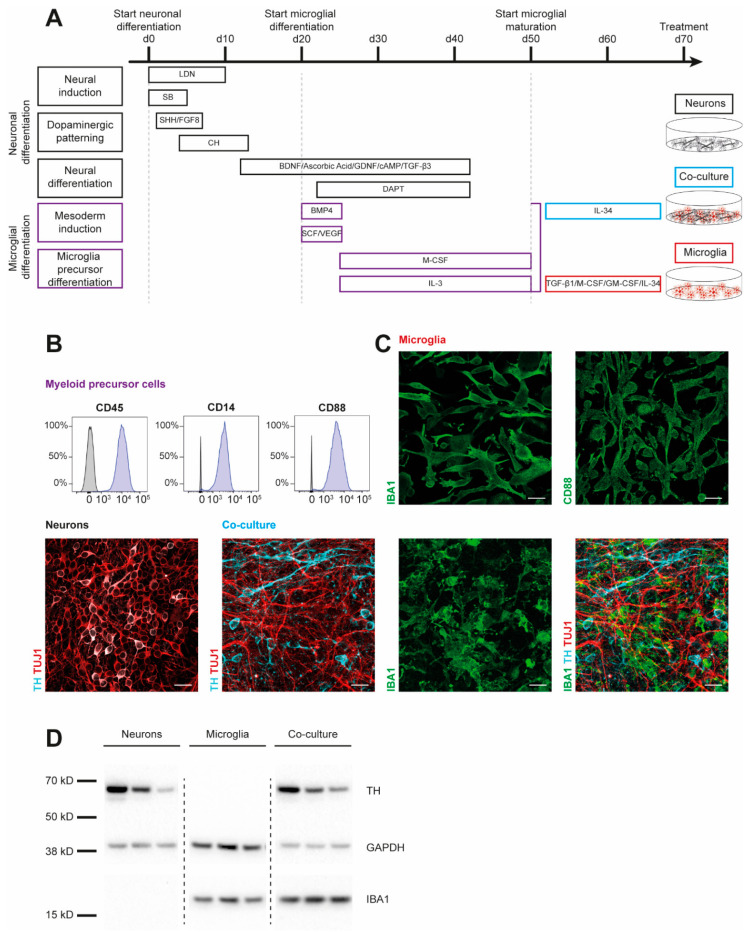
Characterization of iPSC-derived dopaminergic neurons, microglia, and co-cultures. Results are shown for three iPSC lines derived from healthy individuals (SFC086-03-03, SFC089-03-07, and SCF156-03-01). (**A**) Differentiation schemes are shown for microglial (MG) and dopaminergic neuronal (DAN) monocultures, and a combined co-culture (co-DAN-MG). Protocols are based on published methods [15,16,17,18,19]. (**B**) Flow cytometry analysis of the surface markers cluster of differentiation (CD) 14, 45, and 88 in myeloid precursor cells (purple) and iPSC (grey). Images are representative of *n* = 10 independent differentiation experiments. (**C**) Immunofluorescence staining for microglial ionized calcium-binding adaptor molecule 1 (IBA1) (green), CD88 (green), neuron-specific β-III tubulin (TUJ1) (red), and the dopaminergic marker tyrosine hydroxylase (TH) (cyan). Images are representative of *n* = 10 independent differentiation experiments. Scale bar, 25 μm. (**D**) Western blot analysis of IBA1 and TH marker proteins and loading control GAPDH in the three different cultures derived from the three iPSC lines. Whole blots are shown in Figure S4.

To examine cytokine signatures of human dopaminergic neuronal (DAN) and microglial (MG) cultures, we first generated monocultures of three iPSC lines from healthy controls (SFC086-03-03, SFC089-03-07, and SCF156-03-01) using published protocols [15,19]. Briefly, neural induction was achieved by dual-SMAD inhibition. Midbrain floor-plate neuronal precursors were derived from iPSCs upon exposure to activators of sonic hedgehog (SHH) and canonical WNT signaling and were terminally differentiated until day 70 (Figure 1A). To generate myeloid precursors, iPSC-derived embryoid bodies were exposed to macrophage colony-stimulating factor (M-CSF) and IL-3. These myeloid precursors were then treated with IL-34, granulocyte macrophage colony-stimulating factor (GM-CSF), TGF-β1, and M-CSF to derive microglia. Co-cultures of DAN and MG (co-DAN-MG) were generated based on a published method using cortical neurons [17]. On day 50 of DAN differentiation, we added myeloid suspension cells to the dopaminergic neurons and differentiated them into microglia in the presence of IL-34, analogous to the cortical neuron co-culture [17]. Immunofluorescence staining and Western blotting displayed the presence of key markers for the generated cultures (Figure 1B–D). Tyrosine hydroxylase (TH), expressed in dopaminergic cells, and neuron-specific class III β-tubulin (TUJ1) were detectable in DAN and co-DAN-MG. The microglia marker ionized calcium-binding adaptor molecule 1 (IBA1) was detected only in MG and co-DAN-MG. 

### 3.2. MG and co-DAN-MG Cultures but Not DAN Exhibit Cytokine Release Following LPS/IFN-γ Stimulation

**Figure 2:**
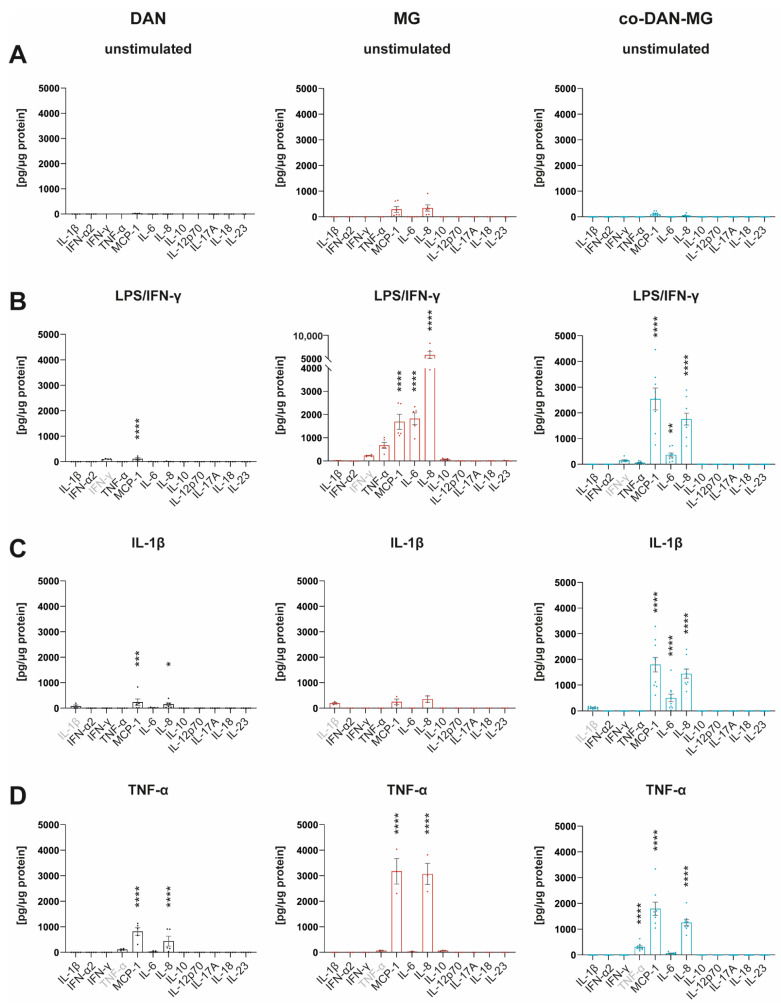
Cytokine profiles of iPSC-derived dopaminergic neurons, microglia, and co-cultures. Simultaneous measurement of 12 cytokines in cell culture media by FACS using Legendplex Human Inflammation Panel 1 under (**A**) unstimulated basal conditions and after stimulation of cells with (**B**) LPS/IFN-γ, (**C**) IL-1β, and (**D**) TNF-α for 24 h. Gray-marked cytokines show measurement of the cytokine used for the respective treatment. Cell culture media of three different lines derived from healthy controls were analyzed: SFC086-03-03, SFC089-03-07, and SCF156-03-01. DAN: *n* = 5 differentiations of 1-3 lines per differentiation experiment, MG: *n* = 2 differentiations of 3 lines each, and co-DAN-MG: *n* = 10 differentiations of 1–2 lines per differentiation experiment. Asterisks indicate significant difference between unstimulated and stimulated cultures by two-way ANOVA followed by Sidak’s multiple comparisons test (* *p* < 0.05, ** *p* < 0.01, *** *p* < 0.001, **** *p* < 0.0001).

Many IFN-γ responses are controlled by the cross-regulation of cellular functions to other cytokines and inflammatory factors [26]. We have used IFN-γ together with LPS to mimic a microglial activation state that is similar to an in vivo situation in which IFN-γ acts in the co-presence of damage-associated molecular patterns (DAMPs) [27]. To compare the cytokine responses with and without this maximal inflammatory activation, we analyzed the media of the three cell lines using a multiplex assay to quantify the levels of 12 different cytokines simultaneously (Figure 2). 

Most of the cytokines were released constitutively at low levels (Figure 2A and Figure S1). MG had a higher baseline number of cytokines secreted compared to DAN and co-DAN-MG. Upon LPS/IFN-γ stimulation, MG showed the strongest response with a 6- to 4464-fold increase (TNF-α, IL-6, IL-8, and MCP-1) followed by co-DAN-MG (IL-6, IL-8, and MCP-1) that displayed an overall reduced secretion (26- to 511-fold increase) whereas for DAN only minor changes were detected for MCP-1 upon treatment (Figure 2B). The reduced secretion of cytokines (TNF-α, IL-6, and IL-8) in co-DAN-MG compared to MG upon treatment with LPS/IFN-γ suggests a co-culture-specific effect on the inflammatory response (Figure S2). Interestingly, there was also a reduced release of cytokines to a similar extend after treatment with TNF-α (Figure S2).

We asked whether the differential cytokine response that we observed among the different cultures is reflected by the expression of the respective receptors and adaptor molecules of the signaling pathway. LPS stimulation of cells expressing cluster of differentiation 14 (CD14) induces Toll-like receptor 4 (TLR4) internalization and myeloid differentiation 88 (MYD88)-dependent signaling from the cell surface, which activates the inflammatory nuclear factor-κB (NF-κB) pathway [28]. Gene expression levels showed significantly higher levels of *CD14*, *TLR4*, and *MYD88* in MG compared to DAN and co-DAN-MG, demonstrating a pattern in accordance with the cytokine response (Figure S3). Notably, gene expression levels of the IFN-γ receptor *IFNR* were comparable between the three different culture types.

### 3.3. IL-1β Stimulates co-DAN-MG but Not MG or DAN Monocultures to Release MCP-1, IL-6, and IL-8

**Figure 3:**
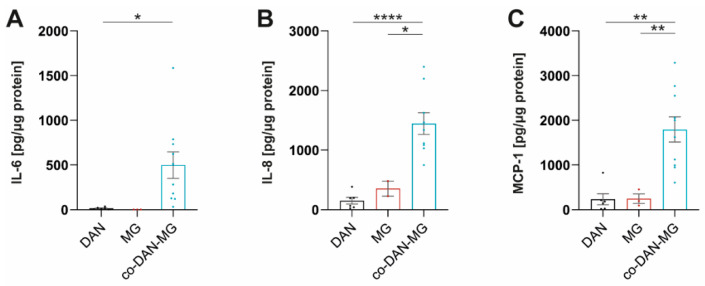
IL-1β stimulates co-cultures but not dopaminergic neuronal or microglia monocultures to release MCP-1, IL-6, and IL-8. (**A**) IL-6, (**B**) IL-8, and (**C**) MCP-1 levels in IL-1β stimulated dopaminergic neurons (DAN), microglia (MG), and co-culture (co-DAN-MG). Media samples were assayed with the Legendplex Human Inflammation Panel 1. DAN: *n* = 5 differentiations of 1–3 lines per differentiation experiment, MG: *n* = 2 differentiations of 3 lines each, and co-DAN-MG: *n* = 10 differentiations of 1–2 lines per differentiation experiment. Cell culture media were taken from three healthy controls: SFC086-03-03, SFC089-03-07, and SCF156-03-01. Mean ± SEM. Asterisks indicate significant difference between different cell models by one-way ANOVA followed by Tukey’s multiple comparisons test (* *p* < 0.05, ** *p* < 0.01, **** *p* < 0.0001).

Treatment of our cultures with IL-1β did not activate inflammatory signaling in MG and led only to a very low-level cytokine release in DAN (Figure 2C). Interestingly, co-cultures displayed a strong response and secreted cytokines at levels comparable to LPS/IFN-γ stimulation. A direct comparison of the three culture types revealed significant differences in the levels of MCP-1, IL-6, and IL-8 (Figure 3). 

Microglia are plastic cells that display region-dependent heterogeneity in the brain regarding their morphology, phenotype, and immune response [29]. Notably, this microglial diversity is partly determined by the local microenvironment and results in transcriptional and phenotypic differences [29]. We tested our cultures for the expression of the *IL-1 receptor* (*IL1R*) gene and found a more than 10-fold increased expression in the co-DAN-MG compared to the monocultures (Figure S3). This suggests that either the underlying differentiation process is co-culture-specific or the local microglial-neuronal microenvironment results in differential inflammatory responses to certain stimulations.

### 3.4. Cytokine Profiling Revealed MCP-1 and IL-8 as the Two Predominant Proinflammatory Factors Released throughout Treatment and Cultures

Treatment with TNF-α demonstrated an increase in MCP-1 and IL-8 levels in all three cultures, including DAN, whereby MG showed the most pronounced cytokine release (Figure 2D). TNF-α interacts with two cognate receptors (TNFRI and TNFRII) expressed by neurons, astrocytes, and microglia throughout the CNS [30] mediating many biological responses. qRT-PCR analysis showed comparable gene expression levels for *TNFRI* between all three cultures (Figure S3), which is in accordance with a response in all cultures. But the observed differences in the levels of released cytokines, when comparing the different cultures with each other, are most likely regulated via additional pathways. 

In summary, cytokine profiling revealed MCP-1 and IL-8 as the two predominant proinflammatory factors released throughout treatment (LPS/IFN-γ, IL-1β, and TNF-α) and cultures. 

### 3.5. Protein Level Changes in PD-Linked Genes in Response to Inflammatory Stimulation

**Figure 4:**
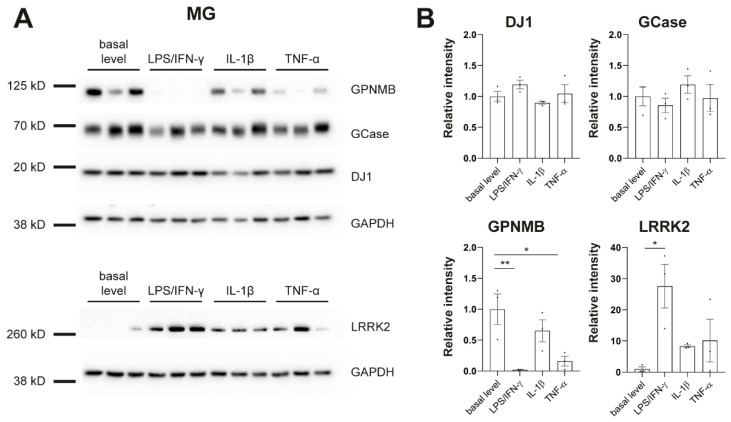
Protein levels of PD-linked genes in response to inflammatory stimulation. (**A**) Western blot analysis shows protein levels of β-glucocerebrosidase (GCase), DJ-1, glycoprotein nonmetastatic melanoma protein b (GPNMB), Leucine-rich repeat kinase 2 (LRRK2), and GAPDH in microglia (MG). (**B**) Signal intensities were normalized to GAPDH. Protein pellets were taken from differentiation experiments of three healthy controls: SFC086-03-03, SFC089-03-07, and SCF156-03-01. Mean ± SEM. Asterisks indicate significant difference between basal level and stimulated cultures by one-way ANOVA followed by Dunnett’s multiple comparisons test (* *p* < 0.05, ** *p* < 0.01). Whole Western blots are shown in Figure S4.

To further phenotype our cultures in regard to PD, we assessed protein levels of genes that have been linked to PD, including *β-glucocerebrosidase* (*GCase*), *DJ-1*, *glycoprotein nonmetastatic melanoma protein b* (*GPNMB*), and *LRRK2* (Figure 4). It has been shown previously that LRRK2 is expressed and upregulated by IFN-γ in iPSC-derived microglia [31]. We replicated this finding. In addition, we found strong downregulation of GPNMB upon treatment with LPS/IFN-γ or TNF-α in MG (Figure 4) which further confirms the link between PD-associated genes and inflammation in human disease models. 

## 4. Discussion

In this study, cytokine profiling of iPSC-derived neuronal and microglial cultures revealed monoculture- and co-culture-specific responses to different inflammatory stimuli. Most importantly, there was a strong co-culture-specific activation upon IL-1β treatment that was not seen in the corresponding monocultures. Moreover, co-cultures responded with a reduced secretion of cytokines compared to MG upon treatment with TNF-α and LPS/IFN-γ suggesting an inhibitory effect within the co-culture system. This shows the unique microenvironment that is present in each of the cultures, which needs to be considered when these cells are used for inflammatory disease modeling. 

We identified IL-8 and MCP-1 as the predominant chemokines released throughout treatment and culture types that were significantly elevated compared to unstimulated conditions. In accordance with that, human fetal microglia produce increased levels of IL-8 in response to LPS, IL-1β, or TNF-α [32]. Notably, both IL-8 and MCP-1 function in the chemotaxis pathway. When neuronal damage is detected by microglia, ramified microglia turn into activated microglia and migrate toward the site of pathology using chemoattractant gradient as a directional cue [33]. IL-8 is a pro-inflammatory cytokine, secreted by neurons, microglia, and astrocytes, that induces neutrophil recruitment to the brain parenchyma where they degranulate and secrete chemoattractants for T lymphocytes [34]. MCP-1 is a chemokine that leads to monocytic chemoattraction into the CNS. Notably, the assessment of CSF levels in different groups of sporadic PD patients revealed that inflammatory markers of the monocyte–macrophage signaling and chemotaxis pathway (Intercellular Adhesion Molecule 1 (ICAM-1), IL-8, MCP-1, Macrophage inflammatory protein-1 beta (MIP-1 beta), and SCF) seem to play a relevant role in PD-associated inflammation [35]. Specifically, increased CSF levels of ICAM-1, IL-8, MCP-1, and MIP-1 beta were associated with decreased scores of mild cognitive impairment and higher CSF levels of neurodegenerative/PD-specific biomarkers in the total PD cohort. Further evidence linking to this pathway comes from *LRRK2* by modulating the expression of genes associated with murine immune cell chemotaxis [36].

It has been reported previously that PD-linked *LRRK2* is expressed and upregulated by IFN-γ in iPSC-derived microglia [31]. We also observed this finding in our cultures. Moreover, we saw downregulation of PD risk factor GPNMB upon treatment with LPS/IFN-γ or TNF-α. Macrophages and microglia have been shown to express high levels of GPNMB [37]. However, the function of GPNMB in inflammation signaling is still unknown. There are several studies suggesting that GPNMB has an anti-inflammatory role by supporting inflammation resolution, but there is also evidence for a pro-inflammatory function of GPNMB. A number of neurodegenerative diseases, including PD [38], are associated with an increase in GPNMB levels in the brain. But GPNMB also seems to have a protective function in neurodegenerative diseases [37]. Increased mRNA levels of *Gpnmb* were reported in one study after 6h of LPS treatment of BV-2 cells (mouse microglia cell line). In contrast, our study shows strong downregulation of GPNMB protein after 24h of LPS/IFN-γ or TNF-α treatment in human MG. Further studies will be needed to clarify the role of GPNMB in neuroinflammation.

## 5. Conclusions

Taken together, these results established cytokine profiles of iPSC-derived cultures, which informs future studies on the reaction to inflammatory stimulation. We found a strong co-culture-specific response to IL-1β treatment indicating a unique microenvironment created by DAN and MG. Moreover, we identified the main types of released cytokines and chemokines in this model system and found a preference for the activation of the chemotaxis pathway in response to all treatments. Finally, we detected protein level changes in GPNMB upon stress in MG, further confirming the link between PD-associated genes and inflammation in human cellular models. 

## Data Availability

Data will be shared with the research community upon request. No code or standardized datasets were generated. This study did not generate new unique reagents. Requests for further information or more detailed protocols should be directed to and will be fulfilled by the corresponding author.

## Supplementary Materials

The following supporting information can be downloaded at: https://www.mdpi.com/article/10.3390/cells12212535/s1, Figure S1: Cytokine profiles of iPSC-derived dopaminergic neurons (DAN), microglia (MG), and co-cultures (co-DAN-MG) under basal conditions; Figure S2: Dopaminergic neuron (DAN) and microglia (MG) co-cultures (co-DAN-MG) show a reduced secretion of cytokines compared to MG upon treatment with TNF-α and LPS/IFN-γ; Figure S3: Gene expression levels of cytokine receptors; Figure S4: Whole Western blots; Table S1: iPSC lines; Table S2: Primers; Table S3: Antibodies.
