# Supplementary material for: Cytokine Profiling in Human iPSC-Derived Dopaminergic Neuronal and Microglial Cultures
Source: Cells. 2023 Oct 27;12(21):2535. doi: 10.3390/cells12212535 (PMC10650774; doi:10.3390/cells12212535)
Supplement: Supplementary file 1 [file cells-12-02535-s001.zip › cells-2669708-supplementary.pdf]

SUPPLEMENTARY MATERIAL – "Cytokine profiling in human iPSC-derived dopaminergic neuronal and microglial cultures"

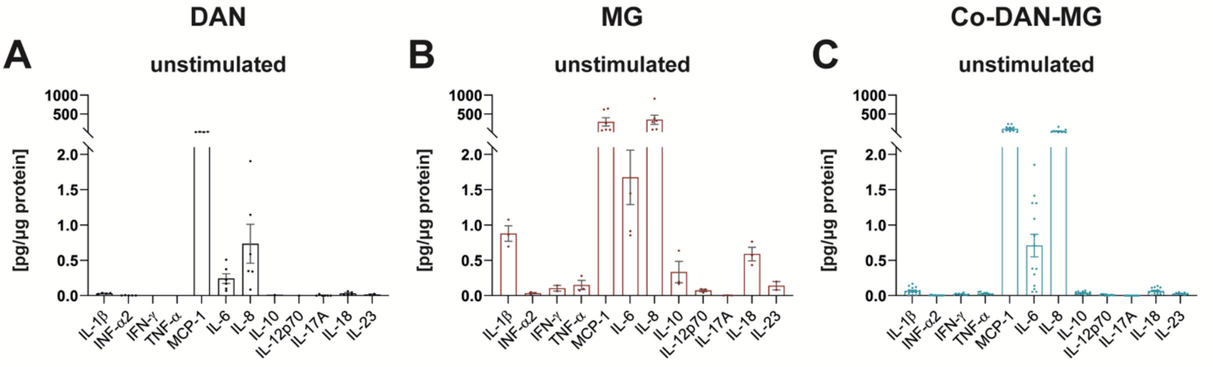

Figure S1. Cytokine profiles of iPSC-derived dopaminergic neurons (DAN), microglia (MG), and co-cultures (co-DAN-MG) under basal conditions. Simultaneous measurement of 12 cytokines in cell culture media by FACS using Legendplex Human Inflammation Panel 1 under unstimulated basal conditions in **(A)** DAN, **(B)** MG, and **(C)** co-DAN-MG. Cell culture media from three different lines derived from healthy controls were analyzed: SFC086-03-03, SFC089-03-07 and SCF156-03-01. DAN: DAN: n=5 differentiations of 1-3 lines per each differentiation experiment, MG: n=2 differentiations of 3 lines each, and co-DAN-MG: n=10 differentiations of 1-2 lines per each differentiation experiment. Mean ± SEM.

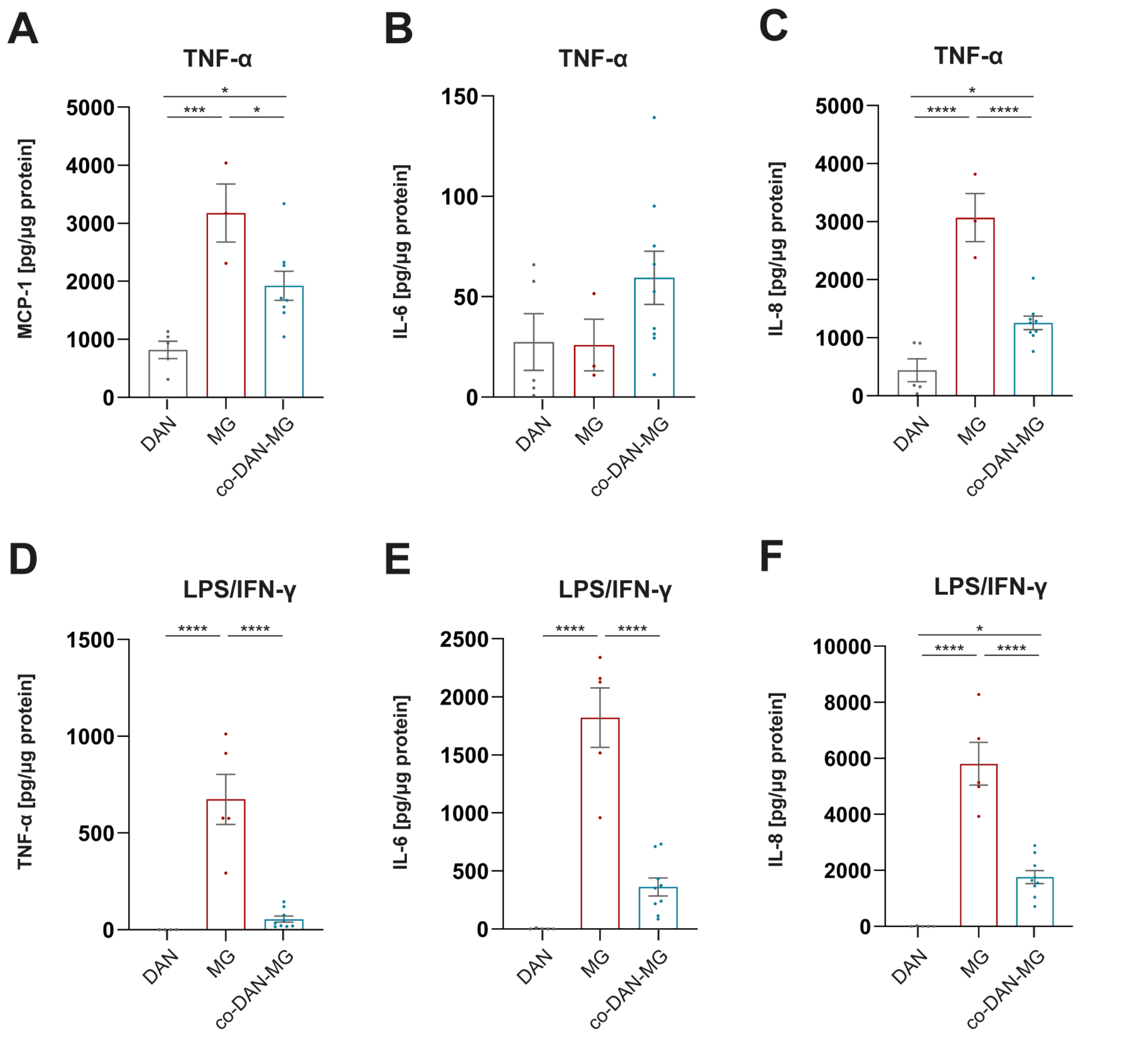

Figure S2. Dopaminergic neuron (DAN) and microglia (MG) co-cultures (co-DAN-MG) show a reduced secretion of cytokines compared to MG upon treatment with TNF-α and LPS/IFN-γ. **(A)** MCP-1, **(B)** IL-6, and **(C)** IL-8 levels in TNF-α stimulated DAN, MG, and co-DAN-MG. Levels of **(D)** TNF-α**,** **(E)** IL-6, **(F)** IL-8 after stimulation with LPS/IFN-γ. Media samples were assayed with the Legendplex Human Inflammation Panel 1. DAN: n=5 differentiations of 1-3 lines per each differentiation experiment, MG: n=2 differentiations of 3 lines each, and co-DAN-MG: n=10 differentiations of 1-2 lines per each differentiation experiment. Cell culture media were taken from three healthy controls: SFC086-03-03, SFC089-03-07, and SCF156-03-01. Mean ± SEM. Asterisks indicate significant difference between different cell models by One-way ANOVA followed by Tukey's multiple comparisons test.

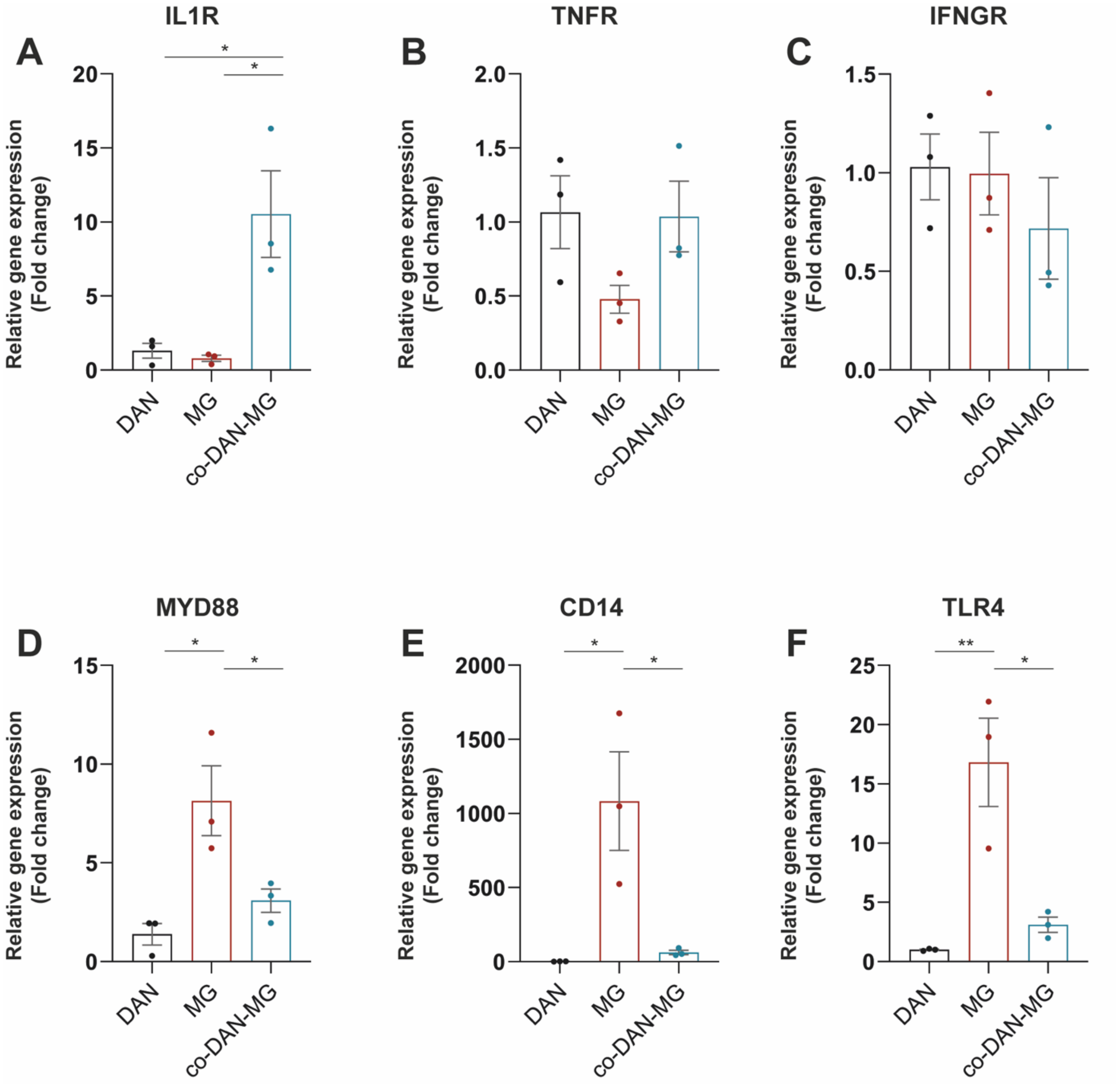

Figure S3. Gene expression levels of cytokine receptors. Relative gene expression analysis of **(A)** *Interleukin 1 receptor* (*IL1R*), **(B)** *Tumor necrosis factor alpha receptor* (*TNFR*), **(C)** *Interferon gamma 1 receptor* (*IFNGR*), **(D)** *myeloid differentiation 88* (*MYD88*), **(E)** *Cluster of differentiation 14* (*CD14*), and **(F)** *Toll-like receptor 4* (*TLR4*) in unstimulated cultures relative to *β-Actin*. n=3 different cell lines (SFC086-03-03, SFC089-03-07 and SCF156-03-01). One-way ANOVA followed by Tukey's multiple comparisons test. Mean ± SEM.

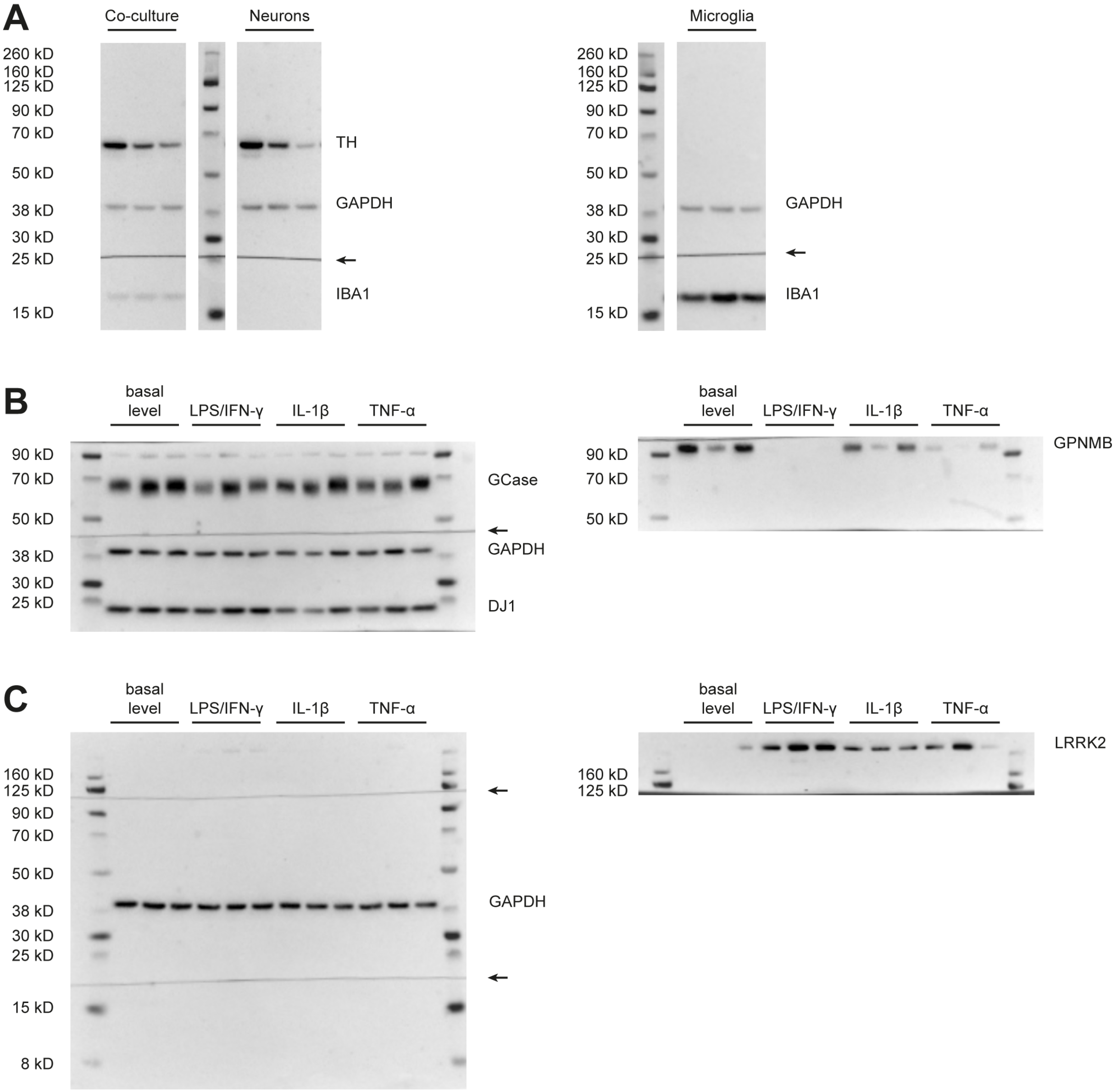

Figure S4. Whole Western blots. Blots show protein levels of **(A)** tyrosine hydroxylase (TH), glyceraldehyde-3-phosphate dehydrogenase (GAPDH), Ionized calcium binding protein (IBA1) and **(B)** β-glucocerebrosidase (GCase), DJ-1, glycoprotein nonmetastatic melanoma protein b (GPNMB), GAPDH and **(C)** Leucine-rich repeat kinase 2 (LRRK2) and GAPDH in stimulated and unstimulated microglia. The blots have been cut and incubated separately with antibodies as indicated by the arrows.

Table S1. iPSC lines

ID of iPSC lines	Sex	Age at biopsy	Reprogramming method	iPSC clone characterization
SFC086-03-03	female	57	Sendai virus	https://hpscreg.eu/cell-line/STBCi052-C
SFC089-03-07	female	64	Sendai virus	https://hpscreg.eu/cell-line/STBCi053-A
SFC156-03-01	male	75	Sendai virus	https://hpscreg.eu/cell-line/STBCi101-A

Table S2. Primers (annealing temperature 58° C)

Gene	Forward primer sequence (5' to 3')	Reverse primer sequence (5' to 3')
Beta-actin	TGAAGTGTGACGTGGACATC	GGAGGAGCAATGATCTTGAT
CD14	CTCTGTCCCCACAAGTTCCC	GGATTCCCGTCCAGTGTCAG
IFNGR1	GAGACGAGCAGGAAGTCGA	TGGAATCGCTAACTGGCACT
IL1R	CGTCCCTGTCCTCTTAACCC	CACACAAGTCCTCCGTCTCC
MYD88	CCGCCTGTCTCTGTTCTT	TCCTCCTCAATGCTGGGT
TNFR1	TCACCGCTTCAGAAAACCAC	TCACTCCAATAATGCCGGTAC
TLR4	CTTTATTCCCGGTGTGGCCA	GCAGGGTCTTCTCCACCTTC

Table S3. Antibodies

Primary antibody	Cat. No.	Dilution	Manufacturer
TH	AB152	1:2000	Millipore, Burlington, MA, USA
GAPDH	2118S	1:30000	Cell Signaling, Danvers, MA, USA
IBA1	Ab178846	1:1000	Abcam, Cambridge, MA, USA
TH	NB 300-110	1:500	Novus Biologicals, Centennial, CO, USA
CD88	344302	1:200	Biolegend, San Diego, CA, USA
LRRK2	Ab133518	1:200	Abcam, Cambridge, MA, USA
GPNMB	Sc-271415	1:1000	Santa Cruz Biotechnology, Dallas, TX, USA
DJ-1	5933S	1:4000	Cell Signaling, Danvers, MA, USA
CD45-APC	21810456	1:100	ImmunoTools, Friesoythe, Germany
CD14-PE	21620144	1:100	ImmunoTools, Friesoythe, Germany
CD88-PE	344304	1:100	Biolegend, San Diego, CA, USA
